# Epidermal growth factor outperforms placebo in the treatment of diabetic foot ulcer: a meta-analysis

**DOI:** 10.12688/f1000research.121712.2

**Published:** 2023-03-10

**Authors:** Fazal Rahim, Xie Yan, Jawad Ali Shah, Nida Bibi, Zafar Ullah Khan, Shah Nawaz, Yao Ming

**Affiliations:** 1 Burn and Plastic Surgery department, General Hospital of Ningxia Medical University, Yinchuan, P.R. China, Yinchuan, 750004, China; 2 School of Biomedical Sciences, Faculty of Health, Queensland University of technology, Brisbane, Australia., Brisbane, 4072, Australia; 3 Tissue Organ Bank & Tissue Engineering Center, General Hospital of Ningxia Medical University, Yinchuan, P.R. China, Yinchuan, 750004, China; 4 Laboratory of Ecology and Evolutionary Biology, and Yunnan Key Laboratory of Plant Reproductive Adaption and Evolutionary Ecology, Yunnan University, Kunming, 650500, China; 5 Department of zoology, Shaheed Benazir Bhutto University, Sheringal 18000, Pakistan., Dir upper, 18000, Pakistan; 6 The second affiliated Hospital, School of Medicine, Zhejiang University 310058, P.R China, Zhejiang, China; 7 Department of infectious diseases, General Hospital of Ningxia Medical University, Yinchuan, P.R. China, Yinchuan, 750004, China

**Keywords:** Diabetic foot ulcer, epidermal growth factor, Placebo, Meta-analysis

## Abstract

**Background:** Diabetic foot ulcers (DFUs) are a life-threatening ailment caused by diabetes. Several growth factors, as well as their various combinations, have shown promising effect in aiding diabetic foot ulcer healing. However, contradictory or paradoxical results are often available, and debates about this issue are ongoing. Therefore, a comprehensive meta-analysis was performed to compare the efficacy and safety of epidermal growth factor (EGF) and placebo in healing diabetic foot ulcers.

**Methods:** The database search included relevant English literature from Cochrane Library, PubMed, Google Scholar, Elsevier, and EMBASE that was published between 2009 and 2021. Inclusion criteria included type 1 and 2 diabetic patients with foot wounds focusing on complete healing rate. Exclusion criteria included combined therapy, non-human studies, reviews, and protocols. To assess the quality of each study, biases regarding random sequence generation, allocation concealment, participant and personnel blinding, outcome assessment blinding and incomplete outcome data were thoroughly identified.

**Results:** Eight randomized control trials comprising 620 patients (337 in EGF group, 283 in placebo group), were included in this meta-analysis. EGF achieved a significantly higher complete healing rate than placebo after four weeks of treatment, with relative risk (RR): 3.04 (0.50, 18.44) and heterogeneity (Chi ^2^ = 6.46, df = 2 (P = 0.04) I ^2^ = 69 %). Notably, the healing frequency in the placebo group was 17%, whereas the healing frequency in the epidermal growth factor group was 34%. Likewise, after eight weeks of treatment, the relative risk and heterogeneity were RR: 2.59 (1.42, 4.72) and (Chi ^2^ =7.92, df= 4 (p= 0.09): I ^2^= 49%), respectively. Moreover, the risk ratio at 12 weeks was RR: 1.01 (0.42, 2.46), and heterogeneity was (Chi ^2^ =8.55, df= 2 (p= 0.01): I ^2^= 77%).

**Conclusions:** Our findings indicate that EGF significantly promotes wound healing, and could be recommended as an effective and safe treatment for DFUs.

## Introduction

Diabetes is a metabolic syndrome, which may be due to the reduced insulin secretion, or defects in insulin function, leading to glucose deficiency, resulting in hyperglycemia. ^1^ Following this, according to international diabetes federation 2017, ^2^ only diabetes was responsible for four million fatalities globally. As a results, 382 million individuals were diagnosed with diabetes in 2013, ^3^ 415 million in 2015, ^4^ 425 million in 2017, ^2^ 463 million in 2019, and is projected to effect 578 million people in 2030. ^5^ Consequently, the average growth rate in diabetes patients from 2013 to 2030 is more than 52%. Increasing tendency of morbidity and mortality is seen in patients with type 2 diabetes, which frequently leads to premature death. ^1^

The three main types of diabetes are type 1 diabetes (T1DM), type 2 diabetes mellitus (T2DM), and gestational diabetes mellitus (GDM). However, the onset of T2DM is more insidious accounting for roughly 90% of all cases. ^6^ Excessive thirst, hunger, weariness, sluggishness, weight loss in type one or progressive weight gain in type two, blurred vision, and passing more urine than normal are the most typical symptoms of diabetes. Hyperglycemia, ketoacidosis, and diabetic coma are the most prevalent acute complications of diabetes. ^5^ In addition, ageing, greater urbanization, genetics, and an obesogenic profile are the primary causes of diabetes. ^7^ People with a family history of diabetes, for example, have a 25% increased chance of inheriting type 2 diabetes from their parents. ^8^ Similarly, monozygotic twins are 90% more likely than heterozygotic twins to acquire T2DM later in life. Moreover, the incidence rates of T1D are also rising, contributing to the increase in diabetes prevalence worldwide. ^9^ However, in depth knowledge for the specific cause of this rise is of paramount importance.

According to the World Health Organisation (WHO) anticipations, diabetic foot ulcers would affect more than 19% of the world’s adult population by 2030. ^10^ Diabetic foot ulcers (DFUs) are wounds in the dermis (the skin’s deep blood vessels and collagen inner layer) that appear below the ankles of diabetic patients. ^11^ It is estimated that DFUs affect 9.1 to 26.1 million diabetic patients each year across the globe. ^12^ Diabetic foot ulcers were found to be prevalent in 6.3% of the world’s population, with North America (13%) having the highest prevalence and Oceania having the lowest (3%). In Asia, Europe, and Africa, the prevalence was 5.5%, 5.1%, and 7.2%, respectively. ^13^ Indeed, diabetes mellitus is one of the most common causes of non-traumatic lower extremity amputation. Approximately 20% of diabetic foot infections that are mild to severe result in amputation. ^14^ The risk of deaths in DFU individuals dramatically raised by 2.5 times when compared to non-DFU patients. ^2^^,^^12^ Insufficient blood circulation due to malfunction of circulatory system significantly increase the incidence of diabetic foot ulcers. Therefore, to treat DFU patients more effectively, it is extremely important to develop a cutting-edge treatment approach.

Several growth factors, including platelet-derived growth factor, fibroblast growth factor, epidermal growth factor (EGF), and peripheral blood mononuclear cells, have showed promise in accelerating ulcer healing in various combinations. ^15^^–^^17^ Inflammation, proliferation, and remodeling are the three stages of wound healing, each of which necessitates the coordination and integration of delicate and complicated biological activities. Chemotaxis, cell proliferation, extracellular matrix deposition, angiogenesis, and tissue reconstruction are all stimulated by the growth factors involved in those biological activities. Several published literature have evaluated the curative effect of topical EGF and placebo on healing diabetic foot ulcers, but there are always contradictions in the evidence to distinguish the true therapeutic effect and safety issues of EGF and placebo in the treatment of DFUs. ^18^^,^^19^ Therefore, a comprehensive meta-analysis was conducted to evaluate the efficacy and safety of EGF and placebo on healing diabetic foot ulcers. The application of EGF, according to our hypothesis, outperforms placebo in facilitating the healing process of DFUs.

## Methods

The meta-analysis was reported according to the preferred Reporting Items for Systematic Reviews and Meta-Analysis (PRISMA) statement. ^20^^,^^29^

### Literature search

**Figure 1.**
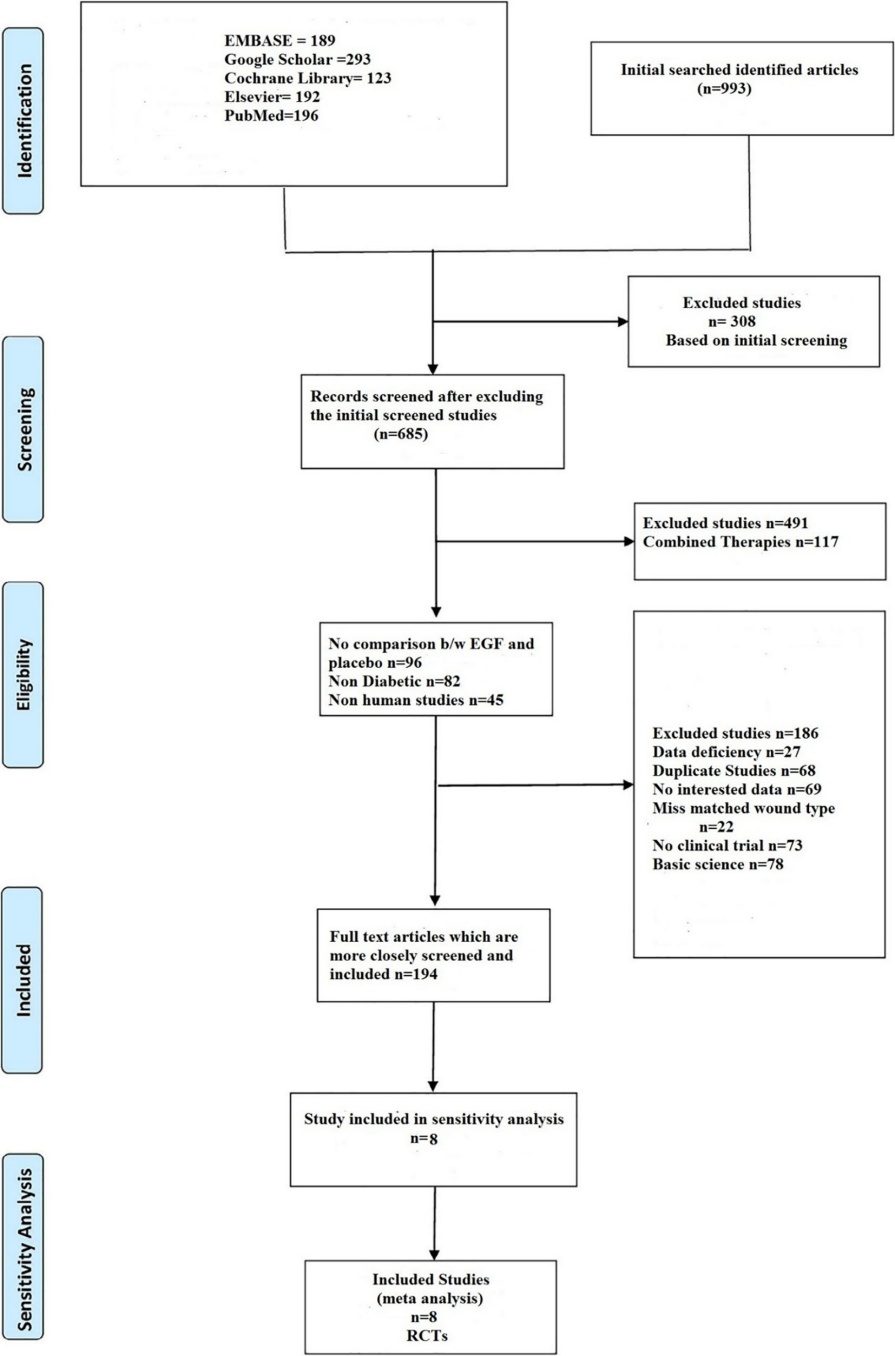
Summary of the studies included.

The search for relevant English literature published between 2009 to 2021 was performed in Cochrane Library, PubMed, EMBASE, and Google Scholar. The specific key terms used in this study includes, Epidermal Growth Factor (EGF), Placebo, Diabetic Foot Ulcer, TDM1 and TDM2. Studies from the reference list were also incorporated in order to find more relevant material. Articles were located and checked on a variety of levels, including title, abstract, and full-text. Final papers that met the inclusion criteria retrieved and included in the study, while those that did not were initially excluded. The full texts of potentially eligible studies were obtained and double-screened for eligibility by two potentials reviewers ( Figure 1). Disagreements were resolved through discussion.

### Eligibility criteria

Inclusion criteria for selecting studies included:

i) Type 1 and 2 diabetic patients with foot wounds.
ii) Literature mainly focused on complete healing time.
iii) Any study design including double-blind, placebo-controlled trial, randomized control trails, retrospective study, prospective study.
iv) Studies comparing epidermal growth factors and placebo in the treatment of diabetic foot ulcers.

Exclusion criteria comprised:

i) Combined therapy
ii) Studies with no reported sample size
iii) Single study of epidermal growth factor or placebo treatment.
iv) Non-human studies, reviews, protocols, and trials.

### Data extraction

The following data were extracted from the eligible studies: initial author’s name, year of publication, location, study time (start-to-end date), number of patients, study design, patient characteristics (average age, sex, etc.), and treatment duration. Authors of articles with insufficient data were contacted for details. Papers were not included in the meta-analysis if the contacted author did not provide the required data. Two independent reviewers extracted the required data.

### Assessment of risk of bias

To assess the quality of each study, the Cochrane risk, a biased evaluation tool in the Review Manager 5.4 programme for RCTs, and the Newcastle-Ottawa Scale (NOS) for cohort studies were employed. We looked at random sequence generation (selection bias), allocation concealment (selection bias), participant and personnel blinding (performance bias), outcome assessment blinding (detection bias), incomplete outcome data (attrition bias), and other sources of bias. In each domain, studies were classified as having a high (red), unclear (yellow), or low (green) risk of bias. The RevMan software regenerated the risk of bias summary table and graph (version 5.4 Copenhagen: The Nordic Cochrane Center, the Cochrane Collaboration, 2014, Denmark). Furthermore, we created a funnel plot to see if there was any publishing bias.

### Statistical analyses

Review Manager 5.4 were used to compile all of the data (RevMan, the Cochrane cooperation, Oxford, UK, RRID:SCR_003581). For this meta-analysis, the heterogeneity between studies was assessed by Cochran (Q) and I ^2^ statistics, which expresses the percentage of variation between studies. I ^2^ was used to evaluate inter study heterogeneity. An I ^2^ value higher than 50% was considered to have statistically significant heterogeneity. We presented dichotomous outcomes as risk ratios (RRs) with their corresponding 95% confidence intervals (CIs). Fixed or random effect techniques are used in meta-analysis, depending on the degree of heterogeneity. The random effect approach is used to combine results when significant heterogeneity is identified in the data, whereas the fixed effect method is used when significant heterogeneity is not detected.

## Results

### Study selection and characteristics

In a systematic search of various electronic databases, a total of 993 articles were identified (EMBASE 189, Google Scholar 293, Cochrane Library 123, Elsevier 192, and PubMed 196). Following an initial screening, 308 studies were ruled out due to their titles. Following that, the remaining 685 articles were carefully screened, with 491 studies being excluded based on full text or abstract, including combination therapies (n = 117), no comparison between epidermal growth factor and placebo (n = 96), non-diabetic (n = 82), non-human studies (n = 45), basic science (n = 78), and no clinical trials (n = 73). The remaining 194 papers were evaluated more thoroughly, and 186 were eliminated due to data gaps (n = 27), duplicate studies (n = 68), content of studies without the desired outcome (n = 69), and wound type mismatch (n = 22). Finally, eight randomized controlled studies were included in the meta-analysis. Figure 1 shows the identified and retrieved articles in the study.

In total, eight randomized control trials (RCTs) that involved a total of 620 patients (337 in the EGF group) and 283 in the placebo group, were included. Except one study from Mexico and another from Cuba, the majority of the studies were from Asia. In these studies, patients received either EGF or placebo intervention, in addition to standard diabetic foot management. The EGF and placebo treatments were administrated by intralesional injection or topical application. All of the patients had type 1 or type 2 diabetes with DFUs. The patient’s ages ranged from 20 to 75 years old. The majority of studies had a follow-up period of 4 to 14 weeks. Table 1 shows the basic characteristics of the included studies.

**Table 1.** Characteristics of the studies that were included.

Author/year	Country	Study design/Number of cases	Intervention Type	Route	Type of DM	Apply frequency	Duration of treatments (weeks)	Number of patients	Age/Ulcer location	Wagner grade	Complete healing/Ulcer duration	Diabetic duration
Fernandez-Montequin, 2019	Cuban	RCT/20	rhEGF 75 μg (n = 53) rhEGF 25 μg (n = 48)	Intralesional Injection	Type 1 and 2	3 times per week	8 weeks	149	65.5/Foot	Grades 3 or 4	60%/4.3 weeks	15 years
Sanjeev Singla, 2014	India	RCT/1	Betadine/Urogastrone (rhEGF) gel 15	Topically	Type 1 and 2	Once every two weeks	8 weeks	50	55-58/Foot	Grade 1 or 2	22.46%/NA	NA
Kwang Hwan Park, 2018	South Korea	RCT/6	EGF 0.005%/Normal saline	Topically	Type 1 and 2	Twice a day	12 weeks	167	56-59/Foot	Grades 1 or 2	62%/NA	NA
Thambi Durai David, 2018	India	RCT/NA	EGF Cream 150 g	Topically	NA	NA	4 weeks	50	25-75/Foot	Grade 1-2	78%/4 weeks	NA
Prabakar, 2016	India	RCT/1	rhEGF/saline	Topically	NA	NA	14 weeks	60	20-70/Foot	Grades 2	75%/NA	NA
Viswanathan, 2019	India	RCT/1	hEGH gel (Regen-D)/Placebo	Topically	Type 1 and 2	NA	4 weeks	50	55-57/Foot and thigh	Grades 1 and 2	45%/NA	NA
Ajay Kundal, 2020	Indian	RCT/1	(EGF)/Conventional Betadine dressing	Topically	NA	NA	8 weeks	60	30-71/Foot	NA	58%/8	NA
Gomez-Villa, 2014	Mexico	RCT/2	rhEGF (75 μg)/Placebo	Intralesional Injection	Type 1 and 2	3 times per week	8 weeks	34	58.6/Foot	Grades 1, 2 and 3	12%/8	16.3 years

Diabetes mellitus (DM); Randomized control trials (RCT); Recombinant epidermal growth factor (rhEGF).

### Risk of bias and quality assessment

**Figure 2.**
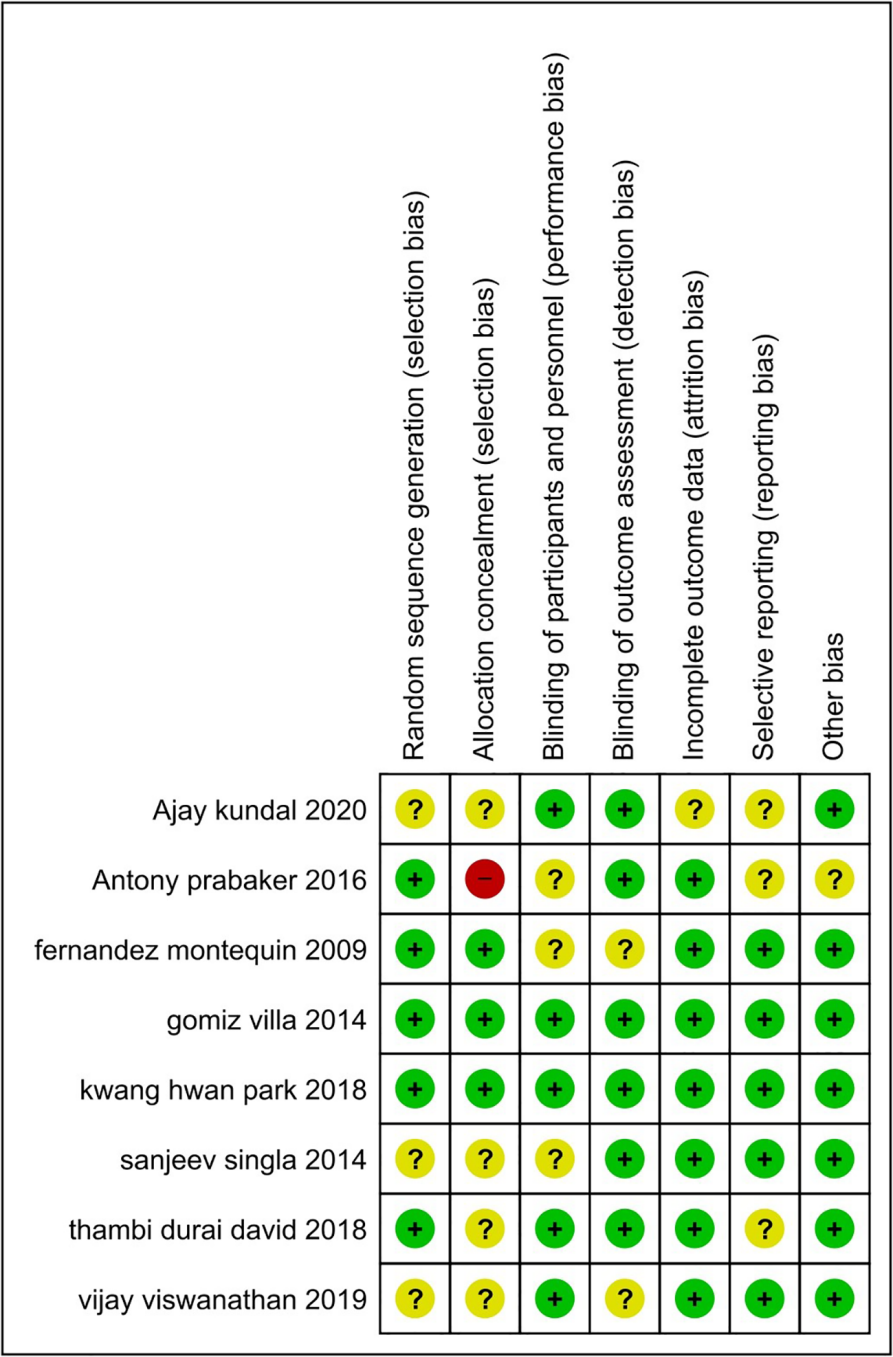
The summary of the risk of bias for each study that was included.

**Figure 3.**
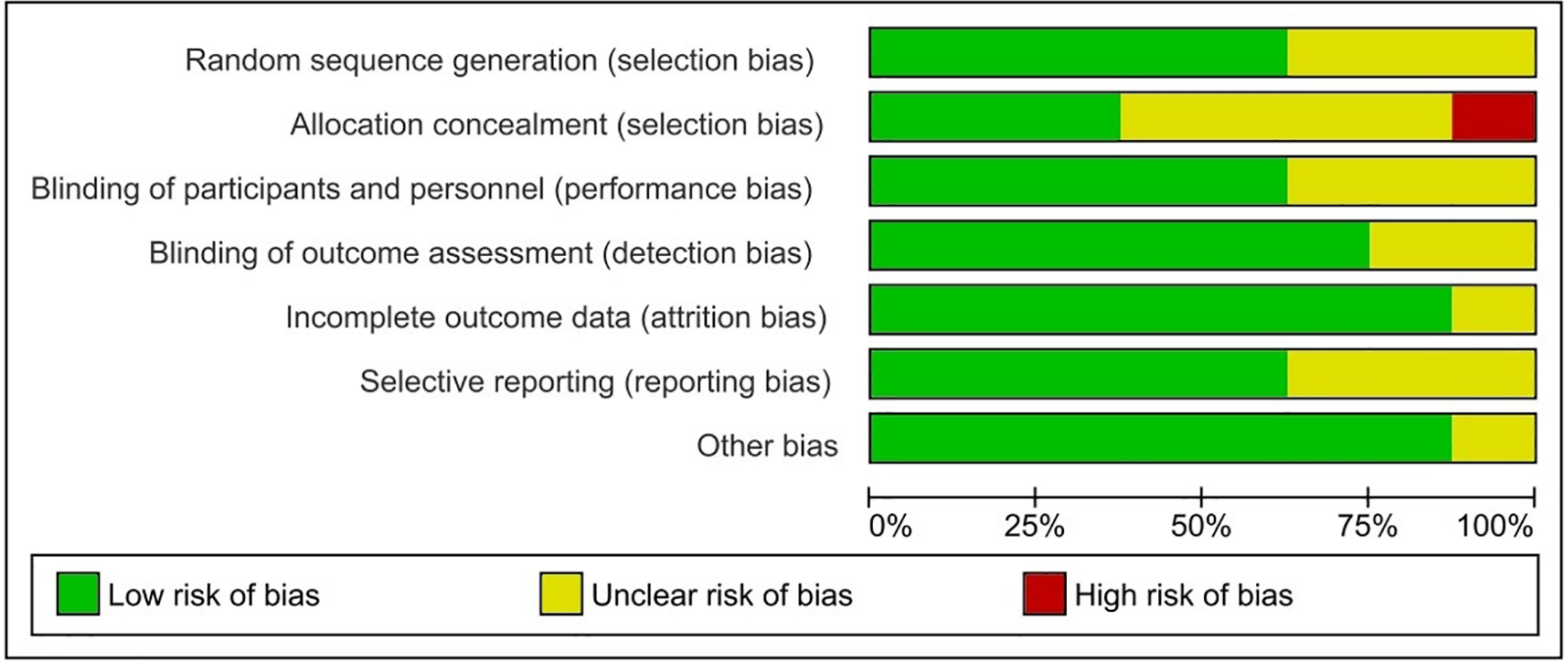
Risk of bias for each study.

**Figure 4.**
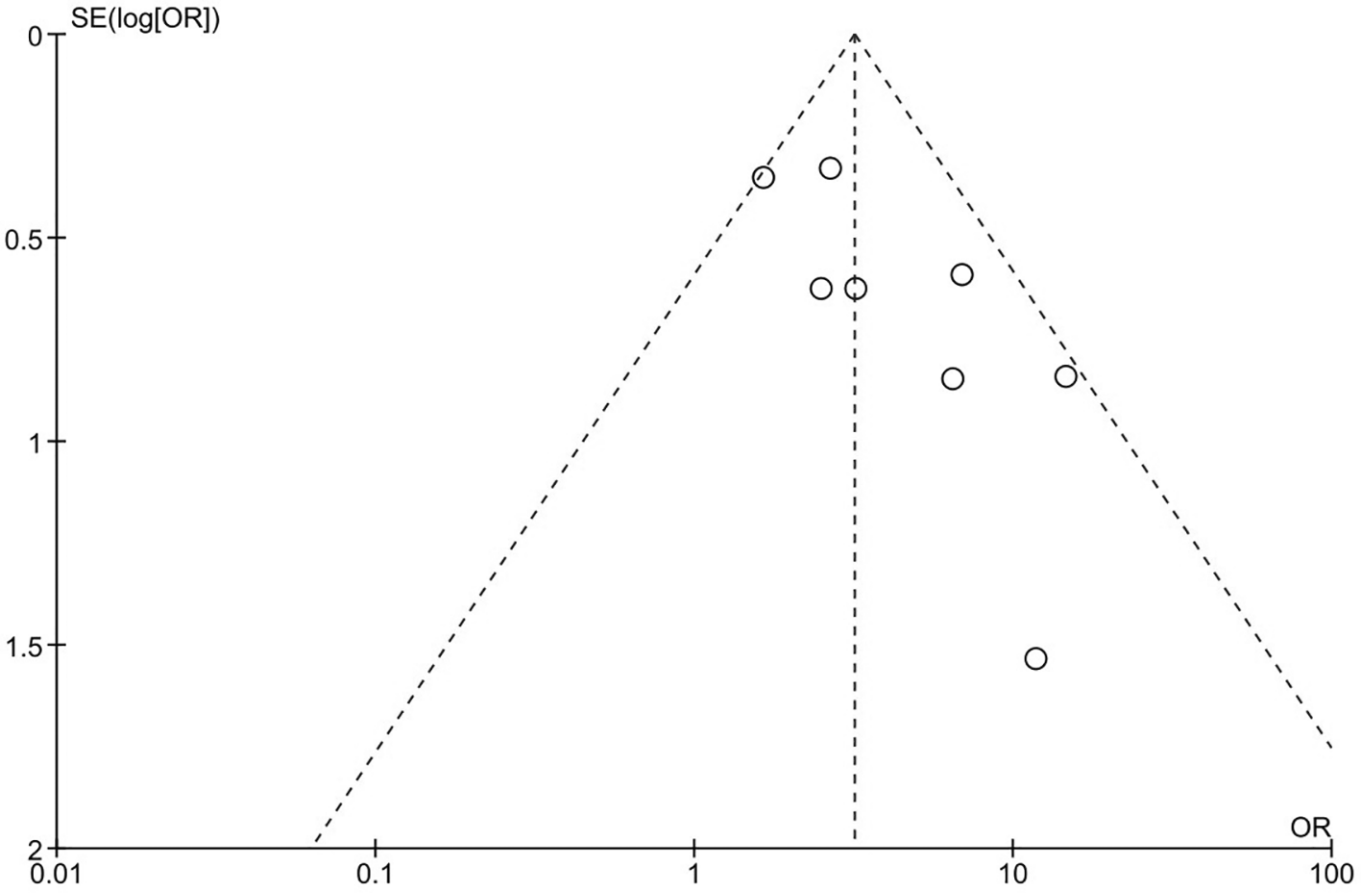
Funnel plot.

Figures 2 and 3 show more information on the risk of bias assessment. The quality evaluation was carried out on a total of eight studies that were included in the qualitative analysis, with the results indicating that the risk of bias was mostly low and unclear. Our risk of bias assessment reported a reasonable quality after adapting the Cochrane Risk of Bias Tool to the Agency for Healthcare Research and Quality (AHQR) standard. The funnel plot exhibited a clear symmetric trend, confirming the absence of publishing bias ( Figure 4).

### Four weeks healing rate of EGF and placebo

**Figure 5.**
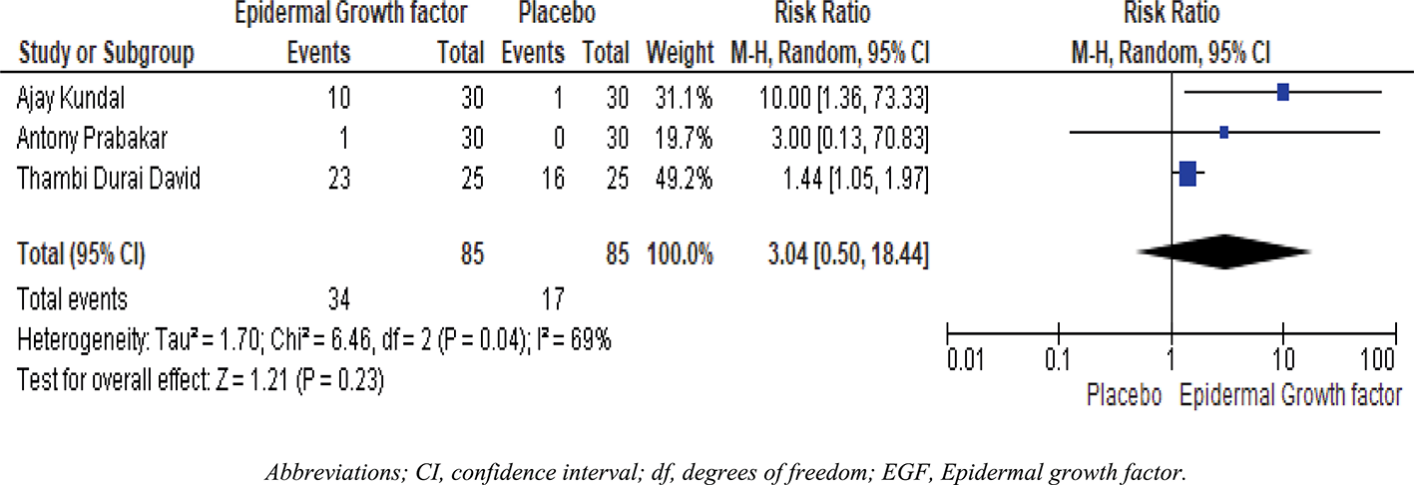
Forest plots and meta-analysis for EGF and placebo group after four-week healing rates. Abbreviations; CI, confidence interval; df, degrees of freedom; EGF, epidermal growth factor.

Statistical analysis results are shown in Figure 5. Three publications including 85 patients were divided into two groups. The epidermal growth factor group recovered significantly faster than the placebo group after four weeks of treatment. For example, with four-week treatment, the healing frequency in the placebo group was 17%, whereas the healing frequency in the epidermal growth factor group was 34%. Furthermore, there is a substantial difference in the healing rate between the epidermal growth factor and placebo groups after four weeks of treatment. Figure 5 shows a significant difference in risk ratio (RR: 3.04, [95 % CI: 0.50, 18.44] I ^2^ = 69%) and heterogeneity (Chi ^2^ = 6.46, df = 2 (P = 0.04) I ^2^ = 69%).

### Eight weeks healing rate of EGF and placebo

**Figure 6.**
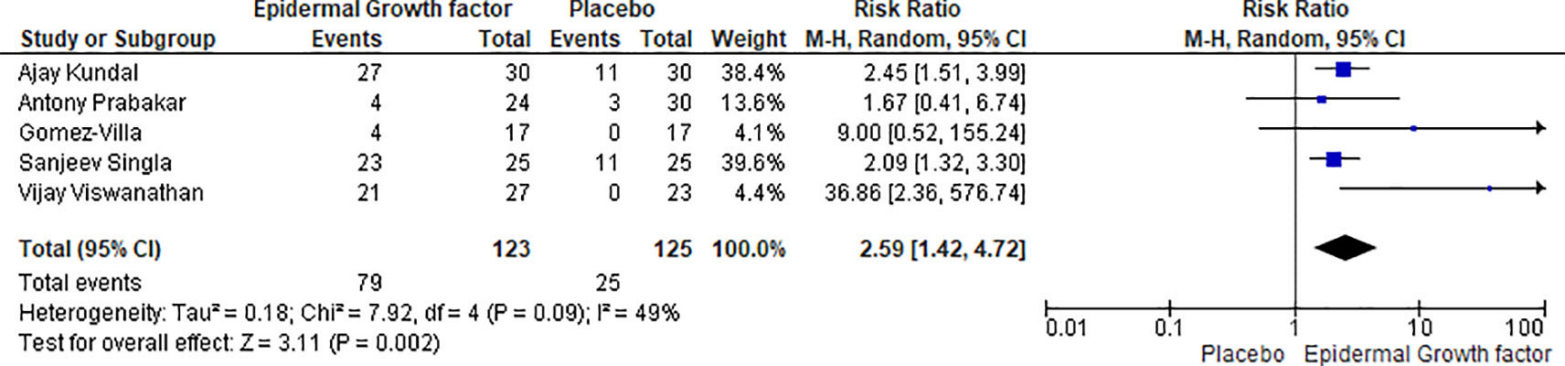
Healing rate of EGF and placebo after eight weeks of treatments. Abbreviations; CI, confidence interval; df, degrees of freedom; EGF, epidermal growth factor.

The eight-week healing rate of epidermal growth factor and the placebo group is compared in five articles. There were 123 patients in the epidermal growth factor group and 125 in the placebo group. After eight weeks of therapy, 79% of patients were recovered with the application of epidermal growth factor, while 25% were recovered in the placebo group, indicating a significant difference ( Figure 6). The proportion of complete ulcer healing with EGF was significantly higher than that of placebo (RR: 2.59, [95% CI: 1.42, 4.72] I ^2^ = 49%) and (Chi ^2^ = 7.92, df = 4 (p = 0.09): I ^2^ = 49%).

### Twelve weeks healing rate of EGF and placebo

**Figure 7.**
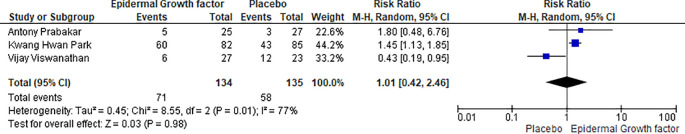
Healing rate of EGF and placebo after twelve-week of treatments. Abbreviations; CI, confidence interval; df, degrees of freedom; EGF, Epidermal growth factor.

A total of three papers compared the healing rate of EGF and placebo groups after twelve weeks of therapy. The EGF group received 134 patients, while the placebo group received 135. There was a clear difference between the epidermal growth factor and placebo groups after twelve weeks of treatment ( Figure 7). The epidermal growth factor group had a healing rate of 71%, while the placebo group had a healing rate of 58%. Furthermore, a significant difference in risk ratio and heterogeneity were noted, with risk ratio (RR: 1.01, [95% CI: 0.42, 2.46] I ^2^ = 77%) and heterogeneity (Chi ^2^ = 8.55, df = 2 (p = 0.01): I ^2^ = 77%).

### Complete healing rate with EGF versus placebo

**Figure 8.**
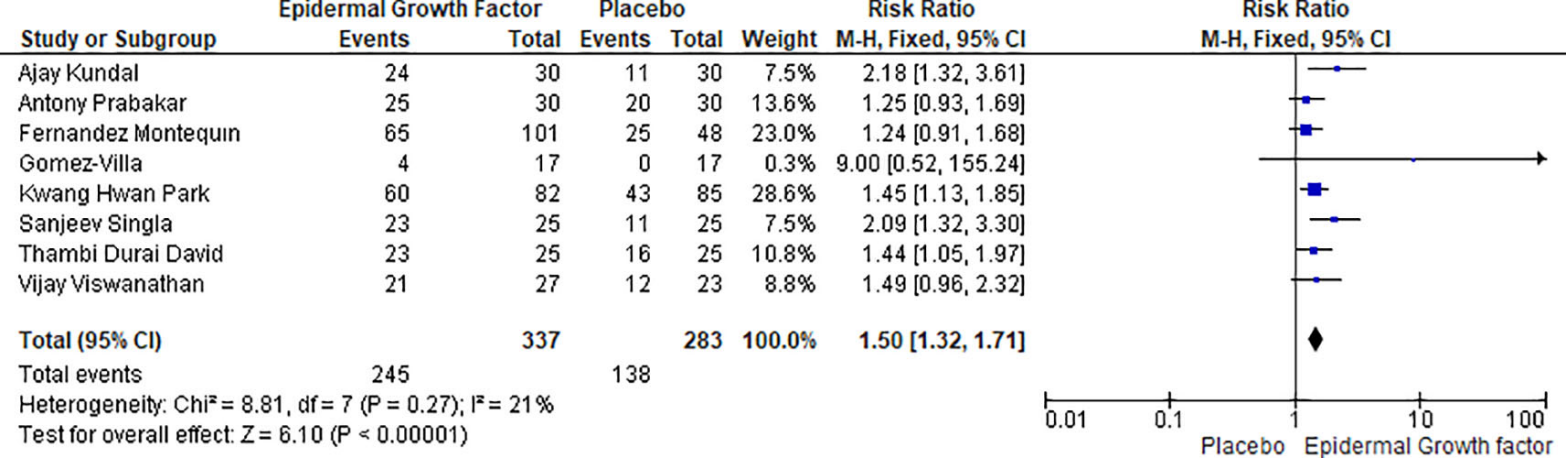
Complete healing rate of EGF and placebo in diabetic foot ulcer patients. Abbreviations; CI, confidence interval; df, degrees of freedom; EGF, epidermal growth factor.

Statistical analysis results for complete healing rate of diabetic foot ulcers in patients treated with EGF versus placebo is shown in Figure 8. The complete healing rate study includes a total of eight publications, with 620 patients demonstrating a complete healing rate; 337 in the EGF group and 283 in the placebo group. For instance, in the epidermal growth factor group a total of 245 (out of 337) healing events occurs, while in placebo group a total of 138 (283) healing events occurs. The risk ratio and heterogeneity were (RR: 1.50, [95% CI: 1.32, 1.71] I ^2^ = 21%) and heterogeneity (Chi ^2^ = 8.81, df = 7 (p = 0.27): I ^2^ = 21%). Overall, the complete healing rate of epidermal growth factor was significantly higher than that of placebo and it could be used as a recommended first line therapy for treatment of DFUs ( Figure 8).

## Discussion

This study was carried out to clarify the efficacy and safety of EGF and placebo in the treatment of diabetic foot ulcer. A total of 620 diabetic patients with foot ulcers were included in the meta-analysis. Several meta-analyses have been published in the last decade ^21^^–^^23^; however, previously published studies frequently share some common limitations. For example, the quality of risk-of-bias was insufficient because of the unknown performance of bias since the patients were not blinded, which could lead to an overestimation of the study’s quality. Another disadvantage is that most studies do not contain any relevant literature at all. Furthermore, past research has employed protocol analysis, ^22^ which has the potential to disrupt the baseline character balance and overestimate treatment outcomes. Therefore, taking all of the inherent limitations into account, a meta-analysis was undertaken based on eight trials that included data from various nations aiming to compare EGF vs placebo alone. The EGF group included 337 patients, while the placebo group includes 283 patients. Our results indicated that the use of EGF significantly improves the healing rate of DFUs relative to placebo group. For instance, the EGF group shows a complete healing rate of 71% while the placebo group shows a complete healing rate of 58.7%. Our findings are similar to those of Quoc Van Phu Bui *et al*., ^22^ who discovered that epidermal growth factor therapy is superior over placebo. Furthermore, Viswanathan *et al*., ^24^ determined a substantial difference between epidermal growth factors and placebo therapy. For example, the EGF group had a complete healing rate of 78%, whilst the placebo group had a complete healing rate of 52%. The most positive conclusion in our study is that EGF improves foot ulcer healing considerably. As a result, as compared to placebo, EGF therapy results in a faster recovery of the wound. The significance of growth factors in wound repair could be one explanation for the remarkable therapeutic impact. EGF promotes epidermal cell proliferation by stimulating glycolysis, mitosis, and protein synthesis. ^25^ By causing inflammatory cells to relocate to ulcer sites, EGF can enhance the wound microenvironment and tissue nutrition. Furthermore, Thambi Durai David *et al*. ^26^ noted that patients treated with EGF had a substantially higher rate of complete healing rates than those treated with placebo i.e. the percentage of total recovery in the EGF and placebo groups was 71.2% and 48.9%, respectively ( Figure 8). Over the course of four weeks, a significant difference was found in cure rates between the EGF and placebo groups, ranging from 3-92% to 0-65%, respectively. Our obtained results are compatible with the findings of the previous meta-analysis. ^21^ For example, Yang *et al*., ^21^ found that, EGF therapy outperformed placebo and the healing rate was 23% in the EGF group and 10% in the placebo group ( Figure 6). Consequently, after eight weeks of therapy, there was a substantial difference between the EGF and placebo groups, with healing rates of 16-92 % and 0-25%, respectively. Following this in a recent meta-analysis, Kumar *et al*., ^27^ found a significant difference between the EGF group and the placebo group, i.e. in the EGF group, complete wound healing was 76%, compared to 20% in the placebo group. Moreover, the average recovery time for totally healed ulcers was eleven weeks ( Figure 7). However, wound healing in the EGF and placebo groups differed significantly after 12 weeks of therapy. The EGF group had a healing rate of 20-73%, while the placebo group had a healing rate of 11-52%. According to previous research by Yang *et al*., ^28^ EGF is far superior to the placebo group, with a complete cure rate of 23.5-95.3% in EGF and 0 to 52.1% in the placebo group. Overall, our findings provide meaningful information to current treatment scenarios of DFUs. As a result, based on current evidence, EGF administration to DFUs is considered as effective and safe.

The most important strengths of our meta-analysis are that; first to our knowledge with eight RCTs this is the most comprehensive meta-analysis on comparing EGF and placebo for DFUs. We also looked for suitable references and emailed the authors for the missing information. Our meta-analysis includes several current clinical studies, which provided more evidence than the prior meta-analysis. This is significant because the number of trials comparing EGF to placebo in the treatment of DFU is currently limited. However, even though publication bias analysis was performed, there are still some potential biases with this review. Some of the literatures included were of poor quality. Although the authors reported that their studies were randomized, the random sequences and blind details were not described in the original articles. The origin of the works did not correspond to a homogeneous recruitment. The economic analysis is important for patients with long diabetic history, especially in developing countries. However, most of the trials didn’t provide cost-effectiveness data. The amount of data on adverse effects was also limited, and hence we could not elicit the most common side effect experienced as a result of EGF and placebo treatment.

## Conclusion

Compared to placebo therapy, EGF significantly accelerate the healing of diabetic foot ulcers at 4-12 weeks of treatment. The EGF has the potential to improve ulcer rehabilitation and speed up wound healing. This conclusion, however, should be approached with caution. More well-designed clinical trials in different populations with long follow-up time are required to further examine the topical EGF therapy in management of diabetic foot ulcer in the future.

## Data availability

### Underlying data

All data underlying the results are available as part of the article and no additional source data are required.

## Reporting guidelines

Figshare: PRISMA checklist for ‘Epidermal growth factor outperforms placebo in the treatment of diabetic foot ulcer: a meta-analysis’. https://doi.org/10.6084/m9.figshare.19928645. ^29^

Data are available under the terms of the Creative Commons Attribution 4.0 International license (CC-BY 4.0).
