# Supplementary material for: Epidermal growth factor outperforms placebo in the treatment of diabetic foot ulcer: a meta-analysis
Source: F1000Res. 2023 Mar 10;11:773. Originally published 2022 Jul 11. [Version 2] doi: 10.12688/f1000research.121712.2 (PMC10587659; doi:10.12688/f1000research.121712.2)
Supplement: Peer review history

# Reviewer report: Reviewer response for version 2

**DOI:** 10.5256/f1000research.145397.r211405

**Published:** 2023-10-19

**Authors:** Poonam Kushwaha

**Affiliations:** 1 Faculty of Pharmacy, Integral University, Lucknow, Uttar Pradesh, India

1. This meta-analysis provides valuable insights into the effectiveness of epidermal growth factor (EGF) in the treatment of diabetic foot ulcers. The comprehensive analysis of eight randomized controlled trials adds to the growing evidence supporting EGF as a promising intervention for diabetic foot ulcers. The study's findings align with previous research, highlighting the significant impact of EGF on accelerating wound healing.
2. The authors have done a commendable job in conducting a rigorous analysis and assessing the risk of bias in the included studies. The quality assessment and the absence of publication bias, as indicated by the funnel plot, contribute to the credibility of the findings. This strengthens the case for EGF as a recommended treatment for diabetic foot ulcers.
3. Diabetic foot ulcers pose a substantial health risk and economic burden, particularly in the face of the rising prevalence of diabetes worldwide. This meta-analysis underscores the urgency of developing effective treatments for diabetic foot ulcers and the potential role of EGF in reducing the associated morbidity and mortality.
4. The study's emphasis on the need for cost-effectiveness data is significant, especially for resource-constrained settings. Future research could delve into the economic implications of EGF treatment in different healthcare systems, shedding light on its affordability and accessibility to a wider population of diabetic patients.
5. One limitation of this meta-analysis is the lack of comprehensive data on adverse effects. Understanding the safety profile of EGF in the context of diabetic foot ulcer treatment is crucial. Future studies should prioritize reporting adverse events to provide a more complete picture of the intervention's overall risk-benefit profile.
6. The consistent findings of this meta-analysis reinforce the importance of growth factors in wound healing, and EGF's role in promoting cell proliferation and enhancing the wound microenvironment. These biological mechanisms provide a scientific basis for EGF's efficacy in diabetic foot ulcer treatment.

Overall, this study contributes significantly to the field of diabetic foot ulcer management. The evidence presented here supports the use of epidermal growth factor as an effective and safe treatment option. This meta-analysis is a valuable resource for clinicians and researchers working to improve the care and outcomes of diabetic patients with foot ulcers.

Are the rationale for, and objectives of, the Systematic Review clearly stated?

Yes

Is the statistical analysis and its interpretation appropriate?

Yes

Are sufficient details of the methods and analysis provided to allow replication by others?

Yes

Are the conclusions drawn adequately supported by the results presented in the review?

Yes

Reviewer Expertise:

Exploration of nanotechnology in enhanced dermal drug delivery.

I confirm that I have read this submission and believe that I have an appropriate level of expertise to confirm that it is of an acceptable scientific standard.

# Reviewer report: Reviewer response for version 2

**DOI:** 10.5256/f1000research.145397.r202888

**Published:** 2023-09-18

**Authors:** Chin Chai Yee

**Affiliations:** 1 School of Pharmacy, Faculty of Health and Medical Science, Taylor's University, Subang Jaya, Selangor, Malaysia

The overall conclusions drawn in the review appear to be generally supported by the results presented in the meta-analysis. The review provides a comprehensive analysis of eight randomized controlled trials (RCTs) by comparing the efficacy and safety of EGF (Epidermal Growth Factor) versus placebo in the treatment of diabetic foot ulcers (DFUs). Here are some constructive comments based on the results and discussion:

1. The review rightly highlights its strengths, including its comprehensive approach with eight RCTs, the efforts made to gather missing information, and the inclusion of recent clinical studies. These strengths enhance the credibility of the meta-analysis and provide more robust evidence compared to prior studies.
2. The review effectively draws comparisons with previous meta-analyses and individual studies, showing that its findings align with existing evidence. This strengthens the case for the efficacy of EGF in DFU treatment.
3. The review acknowledges several limitations, such as the lack of detailed information on randomization and blinding in some studies, heterogeneity in the recruitment criteria, and the absence of cost-effectiveness and adverse effects data. Recognizing these limitations is important as it highlights potential sources of bias and areas where future research can improve.
4. While the review highlights that EGF therapy significantly improves DFU healing compared to placebo, it should emphasize the clinical significance of this improvement. What does it mean for patients in terms of healing time and overall outcomes? Providing more context to the results would enhance the understanding of their practical implications.

Are the rationale for, and objectives of, the Systematic Review clearly stated?

Yes

Is the statistical analysis and its interpretation appropriate?

Yes

Are sufficient details of the methods and analysis provided to allow replication by others?

Yes

Are the conclusions drawn adequately supported by the results presented in the review?

Yes

Reviewer Expertise:

My area of research is lies on biomaterial development and investigating the mechanism of the wound healing effects using bioactive compounds or synthetic drugs.

I confirm that I have read this submission and believe that I have an appropriate level of expertise to confirm that it is of an acceptable scientific standard.
